# Visibility of RF ablation lesions in native T1-weighted MRI reduces with time after ablation

**DOI:** 10.1186/1532-429X-18-S1-P196

**Published:** 2016-01-27

**Authors:** Eugene Kholmovski, Ravi Ranjan, Nathan Angel, Sathya Vijayakumar, Nassir F Marrouche

**Affiliations:** 1 UCAIR, Department of Radiology, University of Utah, Salt Lake City, UT USA; 2 CARMA Center, University of Utah, Salt Lake City, UT USA

## Background

Cardiac RF ablation is a widely accepted procedure for treatment of ventricular tachycardia and atrial fibrillation. Late gadolinium enhanced MRI (LGE-MRI) can be used to assess RF ablation lesions. However, LGE-MRI requires contrast injection and the visibility of lesions and appearance considerably change with time after the injection. Recently, native (non-contrast) T1-weighted (T1w) MRI was proposed to visualize RF lesions immediately post-ablation. The main aim of this work was to study how the visibility and volume of RF lesions in native T1w MRI changes with time after ablation.

## Methods

RF ablations of right and left ventricles of 2 canines were performed according to protocol approved by the local IACUC. RF lesions were created using Cool Flex catheter (St. Jude Medical Inc.) at 30-40 Watts for 60 seconds. Imaging studies were performed on a 3T MRI scanner (Verio, Siemens HealthCare) at 0, 2, and 7 days post-ablation. Native T1w images of whole heart were acquired using 3D respiratory navigated, saturation recovery prepared GRE pulse sequence. Typical scan parameters were TR/TE=3.1/1.4 ms, flip angle of 10 degrees, TI=400 ms, voxel size=1.25x1.25x2.5 mm. Fat saturation was applied immediately before data acquisition limited to 10% of RR interval.

Ablation lesions (enhanced regions) were manually segmented on native T1w images acquired at different time point. Lesion volume was calculated for each ablation (n = 9). Lesion volumes for 2 and 7 days post-ablation studies were normalized by the corresponding lesion volumes from acute (0 day) study. The visibility of ablation lesions in T1w images was quantitatively assessed using Image Intensity Ratio (IIR) metrics. IIR was calculated as a ratio of mean signal of lesion and mean signal of normal myocardium.

## Results

**Figure 1:**
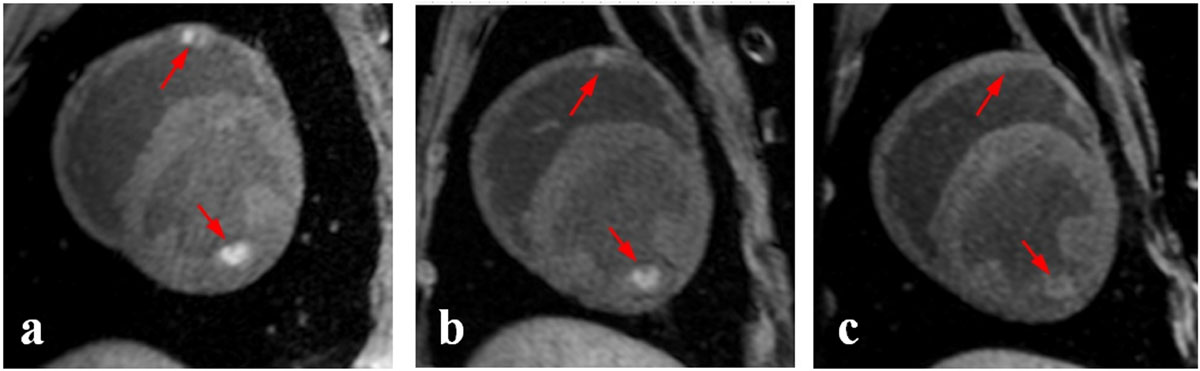
**Representative T1w images of RF ablation lesions acquired (a) acutely, (b) 2 days post-ablation, and (c) 7 days post-ablation**.

**Figure 2:**
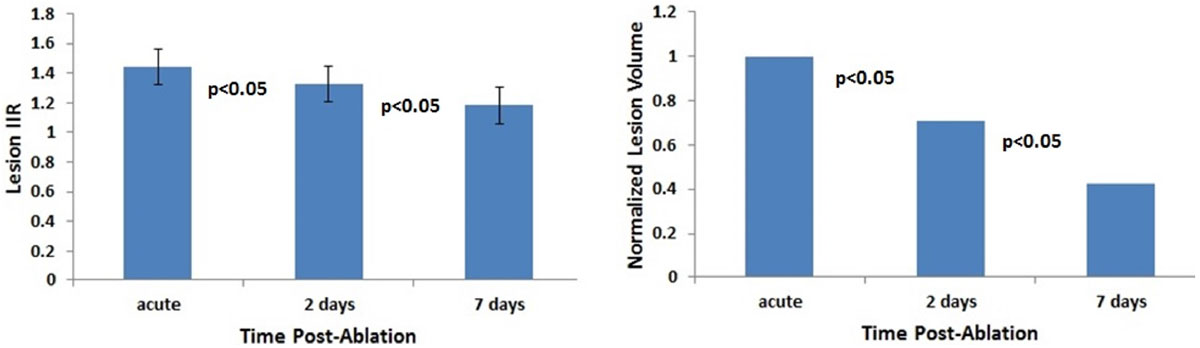
***Left Panel*** **- IIR of RF ablation lesions vs. time post-ablation**. ***Right Panel*** - Normalized Lesion Volume vs. time post-ablation.

Representative T1w images of RF ablation lesions acquired acutely, 2, and 7 days post-ablation are shown in Figure 1. These images clearly demonstrate that visibility of ablation lesions in native T1w images drastically reduces few days after ablation. Quantitate analysis shown that both normalized lesion volume and IIR, decrease significantly (p < 0.05) as early as 2 days post-ablation (Figure 2). This trend continues further with time post-ablation, resulting in the inability to detect many ablation lesions on native T1w images acquired 7 days post-ablation.

## Conclusions

Reduction of T1 relaxation time of RF ablated myocardium has transient nature. The visibility of RF ablation lesions and volume of the corresponding enhanced regions in native T1w MRI drastically reduce with time after ablation. Non-contrast T1w MRI should be performed earlier (< 2 days) after RF ablation to achieve high contrast between ablated and normal myocardium and get an accurate estimate of lesion dimensions.

